# Stress-altering anterior insular cortex activity affects risk decision-making behavior in mice of different sexes

**DOI:** 10.3389/fncel.2023.1094808

**Published:** 2023-01-24

**Authors:** Tianyao Shi, Shufang Feng, Zhonglin Zhou, Fengan Li, Yuan Fu, Wenxia Zhou

**Affiliations:** ^1^State Key Laboratory of Toxicology and Medical Countermeasures, Beijing Institute of Pharmacology and Toxicology, Beijing, China; ^2^Department of Medical Psychology, The Third Medical Center, Chinese PLA General Hospital, Beijing, China; ^3^Department of Pharmacy, Nanjing University of Chinese Medicine, Nanjing, China

**Keywords:** stress, risk decision-making, anterior insular cortex, sex differences, estrogen

## Abstract

Stress can affect people’s judgment and make them take risky decisions. Abnormal decision-making behavior is a core symptom of psychiatric disorders, such as anxiety, depression, and substance abuse. However, the neuronal mechanisms underlying such impairments are largely unknown. The anterior insular cortex (AIC) is a crucial structure to integrate sensory information with emotional and motivational states. These properties suggest that AIC can influence a subjective prediction in decision-making. In this study, we demonstrated that stressed mice prefer to take more risky choices than control mice using a gambling test. Manipulating the neural activity of AIC or selectively inhibiting the AIC-BLA pathway with chemogenetic intervention resulted in alterations in risk decision-making in mice. Different sexes may have different decision-making strategies in risky situations. Endogenous estrogen levels affect emotional cognition by modulating the stress system function in women. We observed decision-making behavior in mice of different sexes with or without stress experience. The result showed that female mice did not change their choice strategy with increasing risk/reward probability and performed a lower risk preference than male mice after stress. Using the pharmacological method, we bilaterally injected an estrogen receptor (ER) antagonist that resulted in more risky behavior and decreased synaptic plasticity in the AIC of female mice. Our study suggested that the AIC is a crucial region involved in stress-induced alteration of decision-making, and estrogen in the AIC may regulate decision-making behavior by regulating synaptic plasticity.

## 1. Introduction

We make decisions all the time. Almost any decision carries some risk that may lead to a loss or an undesirable outcome. Assessing options and making the ‘best’ decision under risk is a critical ability for optimal behavior guidance (Platt and Huettel, 2008; Rangel et al., 2008). As an evolutionary adaptation, humans and animals alike are more willing to avoid risk under uncertainty, a phenomenon known as risk aversion (Tversky and Kahneman, 1981). However, numerous clinical observations have found that excessive risk-seeking behavior is a central symptom of several neuropsychiatric diseases, such as depression, schizophrenia, anxiety, and drug abuse (Rahman et al., 2001; Lee, 2013). Stress is considered a primary risk factor for developing such disorders (Peters et al., 2017), and it can also make us less capable of coping with risky decision-making (Soares et al., 2012). Thus, stress leading to aberrant risk decision-making has received increasing research attention. While the ability to make decisions under risk is crucial to a species’ success, exactly how stress drives behavioral choice is not completely understood.

The anterior insular cortex (AIC), as a key node in the interoceptive network (Wang et al., 2019), can encode both internal and external sensory information and be implicated in many functions related to emotional processing and valence coding (Craig, 2009; Gogolla, 2017). Decisions are influenced by emotional value. Previous brain imaging studies have reported significant activation of the AIC during decision-making under risk (Preuschoff et al., 2008; Werner et al., 2013; Loued-Khenissi et al., 2020), and an insular lesion leads to impaired risk adjustment, further supporting the contribution of this structure to decision processes (Clark et al., 2008, 2014). Additionally, inactivation of the AIC by γ-aminobutyric acid (GABA) receptor agonist can reduce risk-taking during a rat gambling task (Ishii et al., 2012), and microinjection of *N*-methyl-D-aspartate (NMDA) receptor antagonist is sufficient to decrease the social behavior of rat exposed to ethanol during development (Bird et al., 2017). Further studies found that the insula may, through its efferent to the social decision-making network, mediate approach and avoidance responses (Rogers-Carter et al., 2018). This suggests that AIC might contribute significantly to decision-making behavior.

Moreover, stress affects decision-making. Patients with high-stress levels may make bad decisions under risk or even feel tough to make decisions (Miu et al., 2008; Starcke and Brand, 2012). The altered structure and activity of the insular cortex are seen in functional imaging studies of anxiety disorder (Shah et al., 2009; Jeong et al., 2019). The inactivation of the rostral part of the inula induced anxiolytic effects. Conversely, activation caused anxiety-like behavior in rats (Méndez-Ruette et al., 2019). Studies from our laboratory also revealed that the activation of kainate receptor-mediated presynaptic long-term plasticity in AIC contributes to fear and anxiety in mice (Shi et al., 2018). Thus, AIC may be both involved in decision-making and experiencing anxiety. The AIC and basolateral amygdala (BLA) have a strong bidirectional connection and share multiple functions during emotional and cognitive processing. Insula activity has been correlated with several functions that also recruit amygdala activity. Given the prominent functional hierarchical organization of cortico-limbic networks in general, we hypothesized that the insula and amygdala may constitute components of a dedicated cortico-limbic network for effective decision-making. In this circuit (AIC-BLA), the insula is to create and convey a bodily representation to the amygdala, which would be used to modulate and coordinate an emotional response to the stimulus. However, how this circuitry is involved in risk decision-making under stress remains unknown.

Decision-making is influenced by gonadal hormones (van den Bos et al., 2013). Previous results have mostly shown that women are more vulnerable to developing anxiety disorder (McLean et al., 2011), whereas men take more risks than women in various domains (Friedl et al., 2020). Stress may affect decision-making differently in men and women. Therefore, it is crucial to take gender as an essential discriminative factor when studying the interaction between anxiety and decision-making, but the underlying neural mechanism that gender acts as a modulator of decision-making after stress is still unclear.

Estrogen, a sex hormone, is necessary for an extensive spectrum of neural functions, including cognition and emotion (Wharton et al., 2012; Bean et al., 2014). Previous research found that estrogen receptor (ER) null mutant mice have increased anxiety-like behavior with a reduced threshold for the induction of synaptic plasticity in the BLA of mice (Krezel et al., 2001). In the insular cortex of the rat, estrogen plays a role in attenuating middle cerebral artery occlusion (MCAO)-induced excitability (Saleh et al., 2004). For brain-derived estrogen, the rapid modulation of synaptic plasticity may be their essential function (Lu et al., 2019). Given the similarities in synaptic plasticity mechanisms of different regions, we hypothesized that estrogen might play an important role in stress-induced differences in decision-making between males and females. This study also investigated whether estrogen in the AIC regulates risk decision-making after stress. We demonstrated that AIC is involved in the risk decision-making behavior of mice. Stress makes mice take more risks and amplify the behavioral differences between the sexes. Selectively inhibiting AIC-BLA projection can reduce risk-taking. ER antagonist partially increased risk-taking behavior in female mice.

## 2. Materials and methods

### 2.1. Animals

Male or female C57BL6/J mice (8 weeks old; weighing 20 ± 2 g at the beginning of the experiments) were obtained from SPF (Beijing) Biotechnology Co. Unless otherwise specified, mice were housed in groups of four per plastic cage in a 12-h light–dark cycle (from 7 a.m. to 7 p.m.). Food was provided with a standard chow diet, and water was available *ad libitum*. All experimental procedures were approved by the Beijing Institute of Pharmacology and Toxicology. Laboratory animal care was conducted in accordance with the Chinese Veterinary Medicine Association guidelines.

### 2.2. Radial maze-based risk decision-making task for mice

**FIGURE 1:**
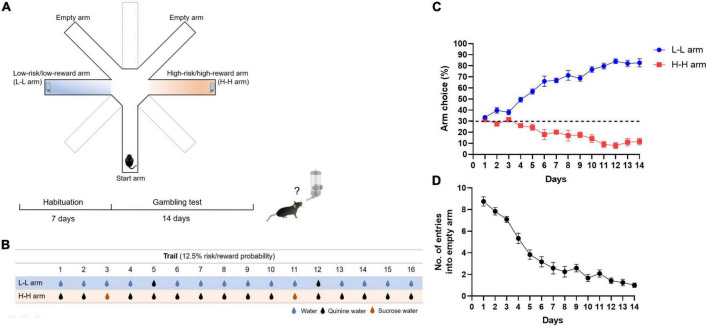
Risk decision-making test for mice. **(A)** Schematic diagram of experimental apparatus and procedure. The apparatus consisted of a start arm and four choice arms, including one low-risk/low-reward baited arm (L-L arm), one high-risk/high-reward baited arm (H-H arm), and two empty arms. A water bottle was glued at the end of the baited arm. **(B)** Risk/reward probabilities of the L-L arm and H-H arm. Under the standard condition, the choice of the L-L arm resulted in small risk (2/16 trials, risk probability: 12.5%) with frequent normal water (14/16 trials, 87.5%). Choice of the H-H arm resulted in infrequent reward (2/16 trials, reward probability: 12.5%) with more significant risk (14/16 trials, 87.5%). Blue droplets: normal water; black droplets: quinine water; red droplets: sucrose water. **(C)** Performance of mice in the gambling test. A dotted line indicates the level of chance for arm choice. **(D)** The number of entries into empty arms.

The experimental apparatus consisted of one star arm and four choice arms (two baited arms and two empty arms), which were modified by a standard radial arm maze (each arm was 40 cm long, 10 cm wide, and 10 cm high; Shanghai Xinruan Information Technology Co., Ltd., China). Unused arms were blocked by a black plastic door. The central platform and arms were light gray. Each end of the arm contained a clear plastic water bottle fixed to the arm ∼3 cm high from the bottom (Figure 1A). The overhead CCD camera allowed detection of the position of animals, and the location of the apparatus was fixed throughout the entire experiment. The apparatus was put in a sound-attenuated testing room.

Before the start of the experiment, each mouse was habituated to and pretrained in the apparatus for 10-min free explorations for 1 week, during which water bottles were placed in all four choice arms that the mice were free to drink. All mice were subjected to 12-h mild water deprivation each day before trials.

In the risk decision-making task, the reward was sucrose water, and quinine water was used for the adverse outcomes. Mice were placed at the end of the start arm and allowed to choose one of the four choice arms when the experiment started. Mice could get different flavors of water in the baited arms and nothing in the empty arms. When a mouse drank water from the bait arm during a trial, it was immediately taken back to the start arm for the subsequent trial to prevent the mice from getting full. When a mouse entered the empty arm, it was taken back to the start arm to continue this trial until it entered the baited arm and drank water. Each mouse was subjected to 16 trials per day, with intertrial intervals of 5 s between each trial, and repeated for 14 consecutive days.

The baited arms contain one low-risk/low-reward (L-L) and one high-risk/high-reward (H-H) arm. In the beginning, we used a 12.5% risk/reward probability as the standard trial condition. In the L-L arm, a mouse has a 12.5% (2/16 trials) probability of drinking quinine water and an 87.5% (14/16 trials) probability of drinking regular water. In the H-H arm, a mouse has a 12.5% (2/16 trials) probability of drinking sucrose water and an 87.5% (14/16 trials) probability to drink quinine water. Quinine water in the L-L arm and sucrose water in the H-H arm were provided randomly at a 12.5% probability during 16 trials per day. L-L arm or H-H arm choice (%) was calculated as follows:


$$Armchoice\left(\%\right)=\frac{T\,o\,t\,a\,l\,n\,u\,m\,b\,e\,r\,o\,f\,e\,i\,t\,h\,e\,r\,b\,a\,i\,t\,e\,d\,a\,r\,m\,s\,e\,n\,t\,r\,i\,e\,s}{16+t\,o\,t\,a\,l\,n\,u\,m\,b\,e\,r\,o\,f\,e\,m\,p\,t\,y\,a\,r\,m\,s\,e\,n\,t\,r\,i\,e\,s}\times 100$$

### 2.3. Single prolonged stress (SPS) procedure

Mice were randomly assigned to a control or stress group. Mice in the stress group were restrained in a 50-ml conical tube with an air vent for 2 h, followed immediately by 20 min of forced swimming in water (20–24°C). Then, after 15 min of recuperation, mice were exposed to ether in a glass desiccator until fully anesthetized without toe or tail pinch response (total time less than 5 min). Mice were immediately returned to their home cages for 7 days without further disturbance until decision-making experimental procedures commenced. Mice in the control group remained in their home cages and undisturbed for 7 days.

### 2.4. Immunohistochemistry

Fluorescence immunohistochemistry was performed as in the previous study (Wei et al., 2021). Briefly, animals were perfused using heparinized saline under deep anesthesia and followed by cold 4% paraformaldehyde in 0.1 M phosphate-buffered saline (PBS). Mice brains were dissected and continuously fixed for 12 h at 4°C. Then, the brains were dehydrated with 30% sucrose in PBS. Then, 40-μm-thick coronal sections were made using a freezing microtome (CM 1950, Leica, Germany), and sections were stored at 4°C in PBS until use. For c-Fos staining, brain slices were made from the mice 1.5 h after the SPS procedure and then incubated with rabbit anti-c-Fos antibody (1:1,000; sys-226-003; Synaptic Systems) for 24 h at 4°C. Then, sections were incubated with Alexa Fluor 488-conjugated donkey anti-rabbit IgG (1:1,000) in PBS for 1 h at room temperature. Finally, the slices were mounted on slides with the fluorescent mounting medium containing DAPI, covered with coverslips, and stored at –20°C for further examination. To visualize and quantify the number of c-Fos positive cells, we use a Zeiss 880 confocal microscope to scan the slices (Zeiss, Oberkochen, Germany). Then, 6–8 different sections from each animal were selected for counting. To examine the effect of CNO treatment on neural activity in the AIC, mice were killed 1.5 h after CNO treatment.

### 2.4. Surgery for viral injection

During surgery, mice were anesthetized with sodium pentobarbital (50 mg/kg). For chemogenetic manipulation, 0.2 μl of AAV-CaMK2α-hM4D(Gi)-mCherry (AAV2/8, 2.57 × 10^12^ genomic copies per ml, OBiO Technology, China) was bilaterally infused through a 33-gauge needle attached to a 10-μl Hamilton syringe at a rate of 0.02 ml/min. The injection site is AIC (AP: 0.74 mm; ML: ± 2.75 mm; DV: 3.75 mm). After injection, the needle was left in place for an additional 10 min to allow total dispersion of the virus. For selective AIC-BLA circuit inhibition, 0.1 μl of anterograde trans-neuronal virus AOV-hSyn-EGFP-Cre (AAV2/1, 5.24 × 10^12^, OBiO Technology) was bilaterally injected into AIC, and 2 weeks later AAV-EF1α-DIO-hM4Di-mCherry (AAV2/8, 9.39 × 10^12^ genomic copies per ml, OBiO Technology, China) was injected into BLA (AP: –1.22 mm; ML: ± 2.80 mm; DV: 4.80 mm). For all experiments, the accuracy of injection coordinates was confirmed by visualization of fluorescent in the injection needle tracks in 40-μm tissue sections.

### 2.5. Slice preparation and electrophysiology

Mice were deeply anesthetized with isoflurane and decapitated. Coronal brain slices (300 μm) containing the anterior insular cortex were cut with a vibratome in oxygenated artificial cerebrospinal fluid (ACSF). For electrophysiological experiments, the brain slices were then transferred to a submerged recovery chamber with oxygenated ACSF at room temperature. This used standard methods for whole-cell voltage-clamp electrophysiology from our previous study (Shi et al., 2018). Briefly, for AIC neuron recording, the excitatory postsynaptic currents (EPSCs) were recorded from layer II/III neurons with an Axon 700B amplifier (Molecular Devices, USA), and the stimulations were delivered by a bipolar tungsten electrode placed in layer V/VI. The recording pipettes (3-5MΩ) were filled with a solution containing (in mM) 124 K-gluconate, 5 NaCl, 1 MgCl_2_, 0.2 EGTA, 10 HEPES, 2 MgATP, 0.1 Na_3_GTP, and 10 phosphocreatine disodium (adjusted to pH 7.2 with KOH). Picrotoxin (100 mM) was invariably used to block GABAA receptor-mediated inhibitory synaptic currents in all experiments. The amplitudes of EPSCs were adjusted between 50 and 100 pA to obtain a baseline. For long-term potentiation (LTP) induction, a TBS protocol was used. Data were discarded if the access resistance changed > 15% during the experiment. Data were filtered at 1 kHz and digitized at 10 kHz.

### 2.6. Statistics

All data are represented as mean ± SEM throughout the text and figures. Primary statistical analyses were conducted using Prism 5 (GraphPad, La Jolla, CA, USA). Differences between groups were analyzed using *t*-tests and one-way or two-way analysis of variance (ANOVA) for multigroup comparisons or repeated-measures ANOVA. In all cases, *P* < 0.05 was considered statistically significant.

## 3. Results

### 3.1. Mice show a preference for low-risk/low-reward arm in a decision-making task

A modified eight-arm maze was used for the risk decision-making task of mice (Figure 1A; Method Details). After 12 h of water deprivation per day, habituated mice were urged to enter the baited arm to find water. It presented the mice with a conflicting choice between sucrose water (high reward) paired with a high probability of quinine water (high risk) and normal water (low reward) paired with a low probability of quinine water (low risk). To find an attractive enough reward and aversive enough condition, preference experiments were performed on different concentrations of sucrose or quinine. It showed that the high concentration of sucrose water did not cause preference but also caused aversive in mice, whereas mice repelled all different concentrations of quinine (Supplementary Figure 1). In this study, 2% sucrose water and 0.5-mM quinine water were chosen as reward or risk factors. The risk/reward probability was set at 12.5% (2/16 trials) at the beginning of the experiment (Figure 1B). Choosing the low-risk/low-reward (L-L) arm may result in randomly encountering bitter water two times in 16 trials but can drink normal water with a high probability (14/16 trials, 87.5%). Choosing the high-risk/high-reward (H-H) arm means that mice can get sweet water with a low probability (2/16 trials, 12.5%), but they are very likely (14/16 trials, 87.5%) to drink quinine water. During 14 days of the gambling test, mice gradually showed a preference for the L-L arm. The L-L arm choice rate reached 82.7 ± 3.7%, while the H-H arm choice was 11.6 ± 3.0% on day 14 (Figure 1C). The number of entries into empty arms, which could be considered an index of the learning ability of mice, dropped rapidly after a few days of task training and is stably maintained (Figure 1D). Arm choice preferences changed with the probability of risk/reward. When the risk probability increased from 12.5% to 50%, the number of L-L arm entries decreased. Conversely, when the reward probability increased from 12.5% to 50%, the number of H-H arm entries increased (Supplementary Figure 2). These results suggest that mice preferred the L-L arm over the H-H arm, similar to healthy humans in the Iowa gambling task (IGT).

### 3.2. Stressed mice take more risk than the control group in the decision-making task

**FIGURE 2:**
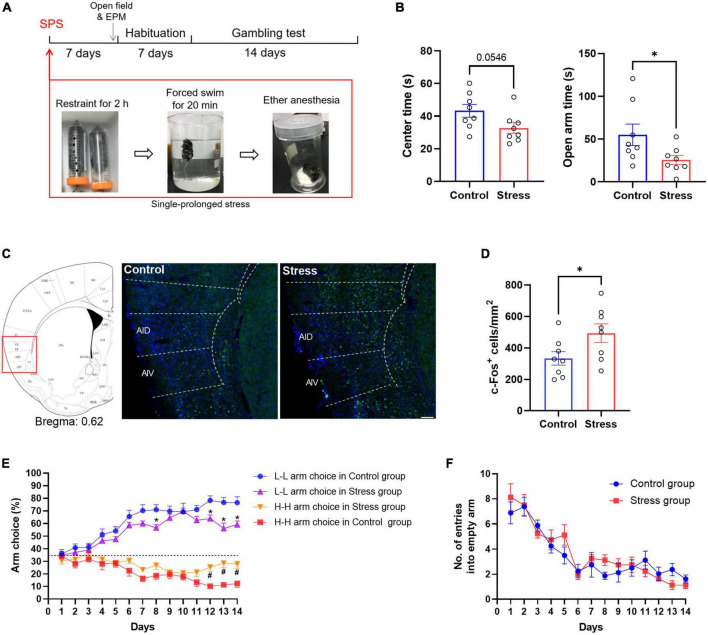
SPS alters the risk decision-making of mice. **(A)** Experimental scheme. Mice were subjected to SPS 7 days before the decision-making task. **(B)** Performance of stressed mice in the open field and EPM test. **(C,D)** Fos immunostained sections of AIC from control and stressed mice and the numbers of Fos+ neurons were analyzed in two groups. *n* = 8 mice per group. Data are presented as means ± SEM. *P < 0.05, Scale bar = 100 μm. **(E)** Performance of stressed mice in the gambling test. A dotted line indicates the level of chance for arm choice. *P < 0.05 vs. the L-L arm choice in control group (*post-hoc* Tukey’s test, *P* = 0.0396 on day 12, *P* = 0.0136 on day 13, *P* = 0.0381 on day 14), ^#^*P* < 0.05 vs. the H-H arm choice in control group (*post-hoc* Tukey’s test, *P* = 0.0092 on day 12, *P* = 0.0070 on day 13, *P* = 0.0220 on day 14). **(F)** Effect of stress on the number of entries into empty arms. There were no significant main effects [treatment: *F*_(1,14)_ = 0.1623, *P* = 0.69; time: *F*_(13,182)_ = 25.30, *P* < 0.05; interaction: *F*_(13,182)_ = 1.134, *P* = 0.33].

To determine the effect of chronic stress on the risk decision-making task, mice were treated with single prolonged stress (SPS), a well-established mice model to mimic behavioral characteristics of post-traumatic stress disorder PTSD (Figure 2A). Stressed mice showed enhanced anxiety-like behavior, such as decreased exploratory time in the center of the open field [*t*_(14)_ = 2.097, *P* = 0.0546, *n* = 8, unpaired *t*-test] and open arm of EPM [*t*_(14)_ = 2.718, *P* = 0.047, *n* = 8, unpaired *t*-test] (Figure 2B). Previous studies from our laboratory revealed that the activation of the anterior insular cortex mediated fear and anxiety-like behavior (Shi et al., 2020, 2022). Significantly increased c-Fos expression was also investigated in AIC after SPS [*t*_(14)_ = 2.194, *P* = 0.0456, *n* = 8, unpaired *t*-test] (Figures 2C, D). The stressed mice gradually performed more likely to choose the H-H arm and less frequently to choose the L-L arm than matched controls in the decision-making task. Repeated two-way ANOVA for the L-L arm revealed significant effects on treatment [*F*_(1,14)_ = 19.81, *P* < 0.001], time [*F*_(13,182)_ = 25.07, *P* < 0.001], and their interaction [*F*_(13,182)_ = 1.561, *P* > 0.05]. For the H-H arm, it also revealed significant effects on treatment [*F*_(1,14)_ = 10.93, *P* < 0.001], time [*F*_(13,182)_ = 8.088, *P* < 0.001], and their interaction [*F*_(13,182)_ = 2.235, *P* < 0.05] (Figure 2E). The number of entries into empty arms did not differ between groups (Figure 2F). These findings suggest that stress-induced changes in AIC activity may play a crucial role in decision-making.

### 3.3. Inhibition of the neural activity of the AIC and AIC-BLA neural projection alters arm choice probability in stressed mice

**FIGURE 3:**
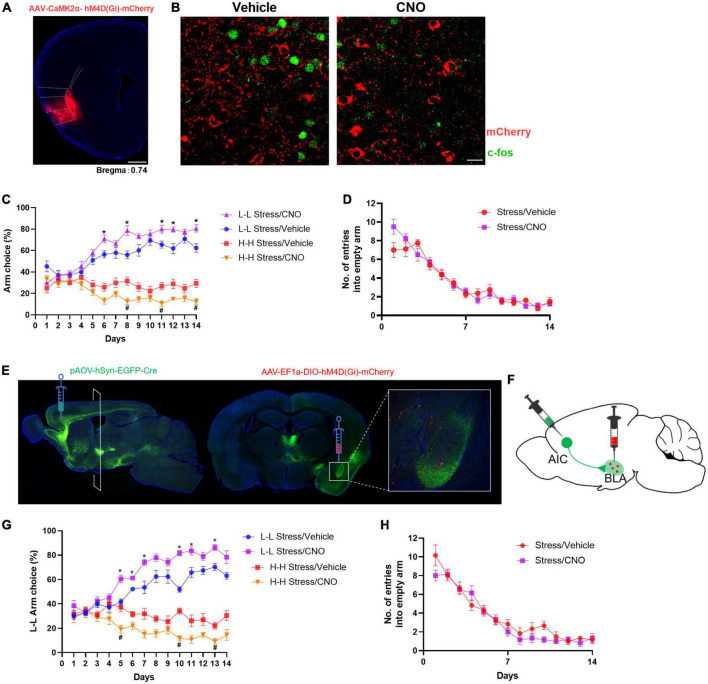
Chemogenetic inhibition of the AIC-BLA circuit disrupts stress-induced abnormal changes in decision-making behavior. **(A)** Diagrammatic representation of the expression of hM4Di-mCherry in the AIC. **(B)** c-Fos expression after CNO treatment in the AIC of hM4Di-overexpressed mice (scale bar: 50 μm). **(C)** Effect of AIC inhibition on stress leads to aberrant decision-making behavior. Repeated-measures ANOVA with Bonferroni correction, *F*_(3,28)_ = 182.3; *P < 0.05 vs. L-L stress/vehicle group (*post-hoc* Tukey’s test, *P* = 0.0475 on day 6, *P* = 0.0087 on day 8, *P* = 0.0495 on day 11, *P* = 0.0370 on day 12 *P* = 0.0191 on day 14), ^#^*P* < 0.05 vs. H-H stress/vehicle group (*post-hoc* Tukey’s test, *P* = 0.0078 on day 8, *P* = 0.0126 on day 11, *P* = 0.0152 on day 14), *n* = 8. **(D)** Effect on the number of entries into empty arms (*F*_(1,14)_ = 0.1889, *P* = 0.6705). **(E)** Schematic representation of the anterograde virus injection strategy. AIC-projecting neurons were found mainly in the basolateral amygdala (BLA). **(F)** Schematic picture of selective labeling of BLA neurons innervated by AIC projection. **(G)** Selectively inhibiting the AIC-BLA circuit can reduce risk-taking behavior of mice. Repeated ANOVA revealed significant effects on treatment [*F*_(1,10)_ = 57.86, *P* < 0.05, n = 6] *P < 0.05 vs. L-L stress/vehicle group (*post-hoc* Tukey’s test, *P* = 0.0041 on day 5, *P* = 0.0094 on day 6, *P* = 0.0338 on day 7, *P* = 0.0001 on day 10, *P* = 0.0366 on day 11, *P* = 0.0097 on day 13), ^#^*P* < 0.05 vs. H-H stress/vehicle group (*post-hoc* Tukey’s test, *P* = 0.0201 on day 5, *P* = 0.0003 on day 10, *P* = 0.0279 on day 13). **(H)** Effect on the number of entries into empty arms (*F*_(1,10)_ = 1.668, *P* = 0.2256).

To determine whether inhibition hyperactivation of the AIC after stress was necessary to alter the risky choice strategy of mice, we used the chemogenetic method and bilaterally injected an AAV vector containing an hM4Di DREADD in the AIC. The gambling test is performed after a 30-min intraperitoneal (i.p.) injection of clozapine-N-oxide (CNO) or vehicle daily. CNO treatment decreased c-Fos expression in AIC (Figures 3A, B). During the 14-day test, CNO-treated mice showed an increased L-L arm choice and a decreased H-H arm choice probability compared with the vehicle group. Meanwhile, there was no significant difference in the number of empty arm entries (Figures 3C, D). This improvement in risk-taking behavior supported a causal relationship between alterations in the activity of the AIC and arm choice after stress.

Furthermore, previous studies have found that the BLA contributes to value computation and the encoding of the valence of positive or negative outcomes (Zhang et al., 2013). The BLA receives prominent projections from the AIC by injecting trans-neuronal virus AOV-hSyn-EGFP-Cre (Figure 3E), and this has also been confirmed in other studies (Lavi et al., 2018; Kayyal et al., 2019). We next searched for the neural circuit pathway of AIC-BLA that supports the risk decision-making behavioral mechanism. After injection of the trans-neuronal virus in the AIC, AAV-EF1α-DIO-hM4Di-mCherry was bilaterally stereotactically injected into the BLA 2 weeks later. The neurons labeled with red fluorescence are innervated by projection from the AIC (Figure 3F). Then, the mice were given i.p. injections of CNO (0.5 mg/kg) or vehicle (PBS) 30 min before the gambling test each day. Compared with the vehicle group, CNO treatment resulted in a gradual increment for L-L arm choice (*F*_(1,10)_ = 57.86, *P* < 0.001; Figure 3G), which further demonstrated that the activation of the AIC-BLA circuit is necessary for stress-induced changes in decision-making behavior. There was no difference in the number of entries into empty arms (Figure 3H).

### 3.4. Male and female mice showed different behavior in the risk decision-making task

**FIGURE 4:**
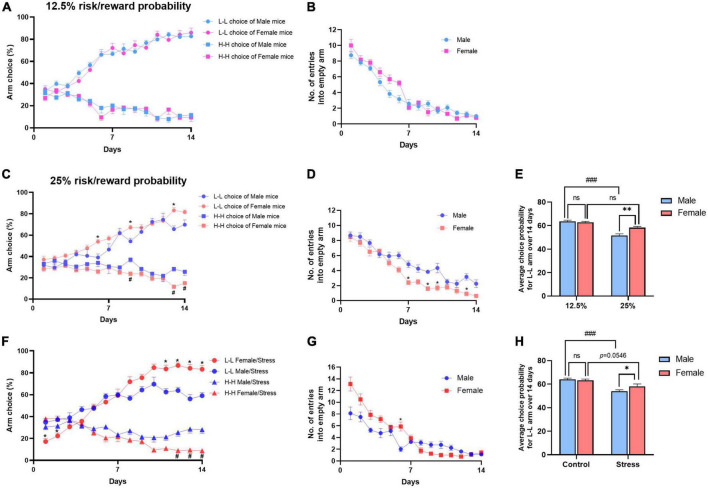
Differences in decision-making between female and male mice. **(A)** Performance of different sex mice in the decision-making test under 12.5% probability. **(B)** Number of entries into empty arms. Not significant [*F*_(1,20)_ = 2.454, *P* = 0.1329]. Dots show choice probability in each session. Repeat ANOVA with Bonferroni correction. Data shown as means ± SEM (*n* = 10-12). **(C)** Performance of different sex mice in the decision-making test under 12.5% probability. Repeated-measures ANOVA with Bonferroni correction. *P < 0.05 vs. the L-L arm choice in male group (*post-hoc* Tukey’s test, *P* = 0.0063 on day 6, *P* = 0.0057 on day 9, *P* = 0.0017 on day 13), ^#^*P* < 0.05 vs. the H-H arm choice in male group (*post-hoc* Tukey’s test, *P* = 0.0019 on day 9, *P* = 0.0001 on day 13, *P* = 0.0479 on day 14). **(D)** Number of entries into empty arms. **(E)** L-L choice rate acquired over 14 days under the standard or double condition. Values are means ± SEM (*n* = 10–12). **P < 0.01 vs. male group under 25% ratio, ^###^*P* < 0.001 vs. male group under 12.5% ratio, n.s., not significant. **(F)** Performance of different sex mice in the decision-making after stress. Repeat ANOVA with Bonferroni correction. Data shown as means ± SEM (n = 8–12). *P < 0.05 vs. the L-L arm choice in male/stress group (*post-hoc* Tukey’s test, *P* = 0.0067 on day 1, *P* = 0.0146 on day 2, *P* = 0.0027 on day 11, *P* = 0.0002 on day 12, *P* = 0.0007 on day 13, *P* = 0.0005 on day 14), ^#^*P* < 0.05 vs. the H-H arm choice in the male/stress group (*post-hoc* Tukey’s test, *P* = 0.0052 on day 12, *P* = 0.0052 on day 13, *P* = 0.0020 on day 14). **(G)** Number of entries into empty arms after stress. **(H)** L-L choice rate acquired over 14 days between the control and stress groups. Values are means ± SEM (*n* = 8–12). *P < 0.05 vs. stressed male group, ^###^*P* < 0.001 vs. control male group, n.s., not significant.

Gender influences decision-making differently. Men tend to make riskier decisions than women (Charness and Gneezy, 2012). In this study, we first observed the decision-making behavior under standard conditions (12.5% risk/reward probability). Male and female mice did not exhibit a significant difference in decision-making behaviors, both showed more preference for the L-L arm and aversion for the H-H arm [for L-L arm, *F*_(1,20)_ = 0.2194, *P* = 0.6446; for H-H arm, *F*_(1,20)_ = 0.0126, *P* = 0.9118, Figure 4A]. There were no differences in empty arm choice ratio between male and female mice [*F*_(1,20)_ = 2.454, *P* = 0.1329, Figure 4B]. When the probability is increased to 25% (4/16 trials), male mice showed higher risk preference, whereas female mice did not exhibit a similar trend compared with male mice [for L-L arm, *F*_(1,20)_ = 12.90, *P* < 0.001; for H-H arm, *F*_(1,20)_ = 18.90, *P* < 0.001, Figure 4C]. There were significant effects on empty arm entries between male and female mice under 25% condition [*F*_(1,20)_ = 23.16, *P* < 0.001, Figure 4D]. The number of entries into empty arms is considered to represent an index of learning and memory of the task rules. These results may suggest that male mice need more time to learn the rules of the task or prefer to spend more time taking risks than female mice. By averaging the L-L choice rate acquired over 14 days, it showed that higher reward did not make female mice more risk-taking. Instead, the female mice chose more the L-L arm than the male mice under the 25% condition [two-way ANOVA, *F*_(1,40)_ = 5.560, *P* < 0.05; Figure 4E]. Furthermore, we evaluated the arm preferences of different sexes after stress. Female mice show less risk-taking than male mice [for the L-L arm, *F*_(1,14)_ = 6.470, *P* < 0.05; for the H-H arm, *F*_(1,14)_ = 20.71, *P* < 0.001, Figure 4F]. Moreover, there were no differences in the empty arm choice ratio [*F*_(1,14)_ = 3.816, *P* = 0.0711, Figure 4G]. Although there was a certain degree of L-L arm aversion, female mice did not show significant risk preference after stress [*F*_(1,34)_ = 1.608, control/female vs. stress/female, *P* = 0.0546, Figure 4H].

### 3.5. Estrogen in the AIC contributes to the risk-taking behavior of female mice

**FIGURE 5:**
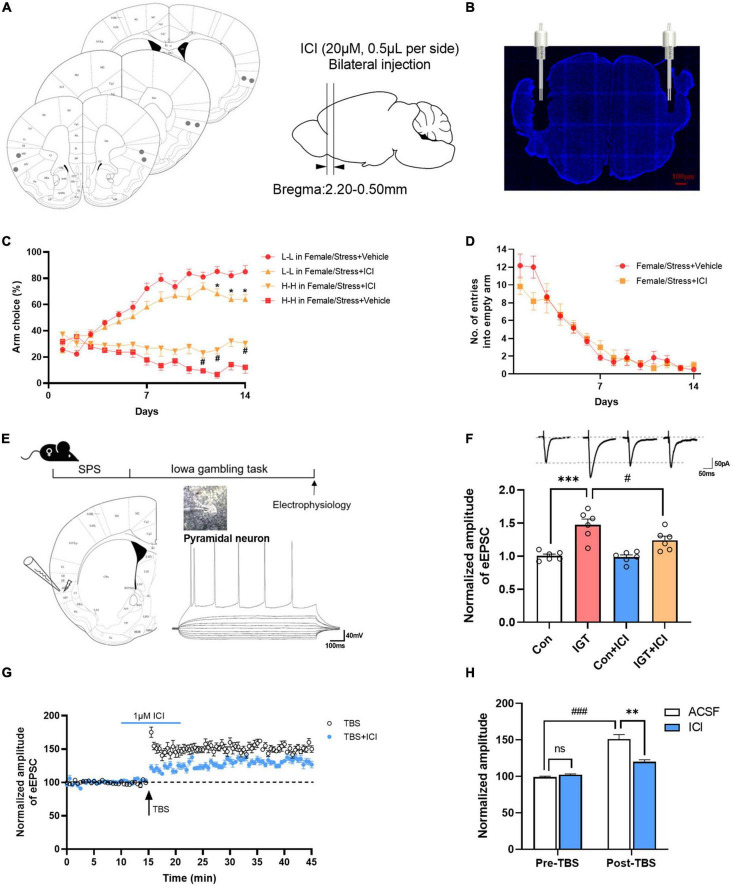
Endogenous estrogen in the AIC contributes to decision-making and synaptic plasticity. **(A,B)** Photograph of coronal section and histological reconstruction of microinjection sites in the bilateral AIC. **(C)** Effect of ICI 182, 780 (ICI) on decision-making task of female mice after stress. Repeated-measures ANOVA with Bonferroni correction. *P < 0.05 vs. the L-L arm choice in the female/stress vehicle group (*post-hoc* Tukey’s test, *P* = 0.0379 on day 12, *P* = 0.0203 on day 13, *P* = 0.0250 on day 14), ^#^*P* < 0.05 vs. the H-H arm choice in the female/stress vehicle group (*post-hoc* Tukey’s test, *P* = 0.0450 on day 11, *P* = 0.0051 on day 12, *P* = 0.0341 on day 14). **(D)** Number of entries into empty arms. **(E)** Diagrams indicating the placement of the stimulating and recording electrodes in the AIC. **(F)** The effects of perfusion of ICI on EPSCs in control (female mice without stress) and IGT group. **(G)** LTP is induced by the TBS protocol (indicated by an arrow). Pre-application of ICI markedly inhibits TBS-induced LTP. **(H)** Summary of the effects of ICI prefusion on LTP recording. ^**^P < 0.01 vs. post-TBS, ^###^*P* < 0.001 vs. pre-TBS, n.s., not significant.

Stabilized estrogen levels are important for regulating and improving effect (Wharton et al., 2012). To test whether the endogenous estrogen in the AIC is also crucial for stress-induced alteration of decision-making behavior, we examined the effects of an intra-AIC injection ICI (ER inhibitor) through cannula during a gambling task (Figures 5A, B). ER antagonist increased the risk-taking behavior of female mice after stress [for L-L arm, *F*_(1,10)_ = 9.234, *P* < 0.05; for H-H arm, *F*_(1,10)_ = 10.97, *P* < 0.001, Figure 5C]. Whereas for mice that did not suffer stress, ICI had no significant effect on the decision-making behavior of normal female mice (Supplementary Figure 3). There was no significant effect on empty arm entries between the vehicle and ICI group [*F*_(1,10)_ = 0.4639, *P* = 0.5113, Figure 5D]. Previous studies have shown that estrogen, as a neuroprotective agent, is essential for synaptic plasticity and normal expression of LTP (Lu et al., 2019). To explore the role of estrogens on synaptic plasticity in decision-making behavior, whole-cell recordings were performed to determine the functional synaptic transmission and LTP in visually identified pyramidal neurons in layers II and III of the AIC. Pyramidal neurons fired repetitive action potentials with frequency adaptation (Figure 5E). Evoked EPSCs were obtained by delivering focal electrical stimulation to layer V/VI of AIC, and neurons were voltage clamped at -40 mV. IGT rapidly potentiated EPSC amplitude within minutes in most of the recorded neurons than the control group [*F*_(3,20)_ = 15.58, ^***^P < 0.001, vs. control group, Figure 5F]. This increment can be partially occluded in the presence of ICI (^#^*P <* 0.05, vs. IGT group), whereas perfusion of ICI alone had no effect on EPSCs. Next, to test the effect of endogenous estrogen on LTP induction, we used TBS to induce LTP. This protocol induced a significant long-lasting potentiation of synaptic responses in the AIC. The LTP amplitude was significantly occluded by ERs antagonist ICI (1 μM) (Figure 5G). Then, we analyzed the amplitude of EPSCs 5 min before TBS (pre-TBS) and 5 min after TBS (post-TBS) to examine the effect of ICI on LTP. ICI perfusion had no effect on the baseline of EPSCs, but significantly suppressed LTP induction immediately after TBS [one-way ANOVA, *F*_(1,40)_ = 18.09, ^**^P < 0.01, vs. ACSF group of post-TBS, Figure 5H]. Together, these findings suggest that estrogen mediated-excitatory synaptic plasticity in AIC plays an essential role in decision-making behavior.

## 4. Discussion

Risk decision-making refers to the judgment and choice of the best solution under the uncertain probability of events occurring. People’s decision-making depends not only on the outcome benefits but also on the psychological satisfaction after the benefits (Loewenstein et al., 2001). Emotional fluctuation has a great impact on this process. Although clinical studies have found that stress alters decision-making and psychiatric patients develop abnormal decision-making behaviors, possible neural mechanisms remain unclear.

There are many paradigms used in the study of human risk decision-making behaviors such as the Cambridge Gambling Task, IGT, and Balloon Analog Risk Task. The most used paradigm for rodents is IGT, a task designed to mimic real-life choices through a conflict between immediate gratification and long-term outcomes (van den Bos et al., 2006). Usually, rodents were placed in a multiarm maze with different probabilities of winning or losing. Rewards or penalties were sugar pellets or quinine-treated food pellets. In most IGT experiments, food deprivation or restriction is necessary to promote the exploratory behavior of rodents. Although the condition of deprivation was strictly controlled, this is still a mild stress for animals with fluctuations in body weight. For animals adaption, the habituation stage had to be appropriately extended before IGT in some studies (Pittaras et al., 2020). In our pre-experiments, hungry mice exhibited more or less anxiety-like behaviors. This may have an impact on our study of how stress affects decision-making. Instead, we used 12-h water deprivation as an induction condition, in which mice only showed small weight changes and no significant anxiety behavior (data not shown). Meanwhile, the habituation stage was also shortened to 1 week. To avoid the development of routines in mice through environmental cues, the apparatus changed orientation randomly across trials. Therefore, the effect of SPS on decision-making behavior can be evaluated more accurately.

The internal affective states affect perceptions of others and shape our social behaviors (Singer et al., 2009). When we make a difficult decision, we feel anxious and uncomfortable in our stomachs. Likewise, when we feel unwell in our bodies, it will also have an influence on our motivational behavior. Previous studies found that high-trait anxious individuals showed activation of the anterior insula with altered error processing during decision-making (Paulus et al., 2004), which was also linked to diminished perceived control (Alvarez et al., 2015). Individuals suffering from depression showed enhanced activity of the anterior insula during pain anticipation compared with healthy controls (Strigo et al., 2013). Our previous studies, using immunohistochemistry and both extracellular and whole-cell patch electrophysiology recording technology, found that anterior insular cortex activation exists in both physical and psychological stress models of mice (Shi et al., 2018, 2020, 2022). In Melissa’s study, they found that NMDA receptors-mediated glutamatergic neurotransmission in the insula plays a prominent role in the restraint stress of rats (Goulart et al., 2022). Moreover, insula activation has been implicated in negative emotions in many imaging studies of humans (Namkung et al., 2017). These seem to indicate widespread activation of the insula after stress. Activation of the anterior insular was also found in heavy drinkers with compulsive alcohol seeking (Grodin et al., 2018). Although not all studies are consistent, for example, damage to the insula abolishes cognitive distortions during risky decision-making tasks (Clark et al., 2014), accumulating imaging studies have highlighted that altered anterior insula activation may be a critical finding in individuals with dysregulated mood (Paulus and Stein, 2010) and anticipation of aversive events (Rogers-Carter and Christianson, 2019). However, neural mechanisms of affective decision-making have rarely been focused on in this brain region. Studies from our laboratory revealed that the activation of AIC is required to induce conditioned fear and anxiety-like behavior in EPM (Shi et al., 2018, 2020).

In this study, significant activation of the AIC was also observed after SPS by Fos-staining. Moreover, as a widely used animal model for PTSD, it biased the decision-making strategy of mice. Chronic stress-caused hypersecretion of glucocorticoids is a main factor of psychopathologies ranging from mood dysfunction to cognitive impairments. Previous studies have mainly found limbic–cortical circuits involved in coordinating the behavioral responses to stress and execution of actions, particularly the mPFC and amygdala have attracted much attention from researchers interested (Dias-Ferreira et al., 2009; Soares et al., 2012). An earlier lesion study compared the different roles of the insula, amygdala, and ventromedial prefrontal cortex in a decision-making task. Although each neural region seems to contribute to the distinct process of decision-making, the patients with the amygdala and insula showed more domain-specific decision deficits (Weller et al., 2007, 2009). The insula has been proven to be an anatomical integration hub with heavy connectivity to an extensive network of cortical and subcortical brain regions serving sensory integration and emotional process (Gogolla, 2017). Recently, studies also proved that the BLA→AIC pathway participated in rewarded contextual memory (Venniro et al., 2017) and tracking-specific Pavlovian devaluation sensitivity (Keefer et al., 2022). The AIC→BLA pathway is involved in the modulation of fear and anxiety (Shi et al., 2020). Here, we inhibited the activity of AIC and further selectively inactivated the AIC→BLA projection neurons by the chemogenetic method, both demonstrating that the AIC and its pathway are crucial for the decision motives of whether to take a risk.

Men and women behave differently when making decisions (Derntl et al., 2014). Men usually escalate risky decision-making more than women under pressure, whereas stress has no effects or even decreases risk-taking in women (Lighthall et al., 2012; Kluen et al., 2017). Sex hormones are likely to be one of the reasons for this difference. Depending on different decision-making tasks, men and women sometimes behave differently. In some studies, men but not women tend to make more risk-averse decisions after acute psychological stress (Fairchild et al., 2009). In our study, we used IGT as a laboratory-based decision-making task for mice. Under standard conditions, male and female mice show no difference in decision-making strategy. When reward probability doubled, male mice are more likely to choose a high-reward/high-risk arm than female mice. Furthermore, after chronic stress, female mice showed higher risk aversion than males.

Estrogen, a steroid hormone, involves cognitive functions and emotion (Gasbarri et al., 2012). A large number of studies have shown that there are gender differences under stress and estrogen may play an important role in the regulation of stress response. Low estrogen levels can make women more vulnerable to trauma at some points in their menstrual cycles, while high levels of the female sex hormone can partially protect them from emotional disturbance. Moreover, women or female rodents showed less fear response to the negative stimulus when estrogen was high than when it was low. The estrous cycle also modulates the spatial memory of mice under stress (Lebron-Milad and Milad, 2012). Therefore, regulating estrogen levels might one day be used to help prevent post-traumatic stress. In addition to the regulation of the stress response, the difference in the decision-making behavior of men and women is also closely related to the level of estrogen. Most human studies have focused on the menstrual cycle’s effects on decision-making (Ambrase et al., 2021). Men seemed more biased toward maximizing rewards while women prefer frequent but smaller rewards. The ability of estradiol to influence emotional and cognitive functioning is largely by modifying gene expression through classical genomic as well as non-genomic membrane-associated receptors in the human brain (Marino et al., 2006). For example, estradiol can bind to G protein-coupled receptors and directly trigger mitogen-activated protein kinase and phosphatidylinositol-3 kinase cascades (Mannella and Brinton, 2006). Besides the activation of its own receptors, estrogen hormones can also act on other receptors, including GABA, NMDA serotonin, and dopamine receptors, thereby pivotally modulating synaptic plasticity and neurotransmission in the specific location of the brain (Barth et al., 2015). Evidence from animal studies suggested that estrogen acutely potentiates excitatory synaptic transmission and facilitates the formation of LTP in the hippocampus involved both presynaptic and postsynaptic mechanisms (Smejkalova and Woolley, 2010). In the anterior cingulate cortex of mice, ERα and ERβ were found to have colocalization with NMDAR subunit and rapidly enhanced NMDA-evoked current by estradiol perfusion (Xiao et al., 2013). Therefore, the synergistic effects of estrogen on cognitive and emotional function may underlie the association of social decision-making and depression in women under psychosocial stress (Albert and Newhouse, 2019).

In the AIC, a structural fMRI study reported gender-related differences in the insula in boys suffering from PTSD symptoms, showing larger volume and surface area than their healthy male counterparts. In contrast, a smaller average volume and surface area of the insula could be detected in girls with PTSD compared with control girls (Klabunde et al., 2017). Although these structural changes cannot be determined as a direct result of sex hormone differences, long-term estrogen administration did alter the functional connectivity of the insula in another study (Reed et al., 2022). Furthermore, Charlotte and her colleagues also revealed that greater increases in estrogen levels across the menstrual cycle led to less impulsive decision-making (Smith et al., 2014). Although some reviews suggested that estrogen can attenuate emotion processing and supports mood regulation in women, the effects of estrogen in women with risk decision-making, in whom insula activity is likely altered, remain less understood. Here, we implanted cannulas and injected ER antagonists directly into the AIC before each decision trial. The results showed that female mice made more risky choices than vehicle mice after stress. This may be due to the inhibition of ER -mediated synaptic transmission in AIC. Therefore, moderate estrogen is vital for maintaining normal cognitive and emotional function. It is also confirmed in human studies that even acute and short-term changes in estrogen might impact brain functions (Craig and Murphy, 2007). Homeostasis of endogenous estrogen is key to making appropriate decisions under stressful conditions.

## 5. Conclusion

In this study, we examined the effects of a single prolonged stress exposure on the risk decision-making task of male and female mice. Using the chemogenetic methods, we observed the role of the AIC-BLA circuit in decision-making, and further, by pharmacologically inhibiting ERs, we investigated the possible reasons for the different decision-making strategies between male and female mice caused by sex hormones. Further research is still needed to elucidate the role of estrogen on the mechanistic underpinnings of AIC activation and risk decision-making after stress. Moreover, there are some things that could be more consistent between animal data and human research, which would require us to further examine different subregions of the insular cortex using more advanced neural manipulation technology.

## Data availability statement

The original contributions presented in this study are included in the article/Supplementary material, further inquiries can be directed to the corresponding authors.

## Ethics statement

This animal study was reviewed and approved by Beijing Institute of Pharmacology and Toxicology.

## Author contributions

TS and SF were involved in designing the study and writing the manuscript. TS and ZZ carried out all experiments and analyzed the data. WZ helped in revising the manuscript. FL and YF helped in performing the behavioral testing. All authors read and agreed to the published version of the article.

## Supplementary material

Supplementary Figure 1 **(A)** Effect of different mass fractions of sucrose solution on sucrose preference. During the habituation stage of the risk decision-making test, water and sucrose solutions were placed separately in the baited arm. The consumption volume was calculated in 10-min free exploration. **(B)** Percentage of sucrose consumption to the total solution consumed (%). **(C)** Effect of different mass fractions of quinine solution on quinine aversion. **(D)** Percentage of quinine consumption to the total solution consumed (%). **P* < 0.05, ^**^*P* < 0.01, ^***^*P* < 0.001 (two-way ANOVA with Bonferroni correction).

Supplementary Figure 2 **(A)** Effect of changing the probability of risk/reward on arm choice ratio in the mice gambling test. The risk and reward probability were increased from 12.5% to 50% simultaneously. Values are means ± SEM (*n* = 12). Repeated ANOVA revealed significant effects on risk/reward probability *F*_(5,66)_ = 137.7, *P* < 0.001, time *F*_(66,858)_ = 2.029, *P* < 0.001, and their interaction *F*_(65,858)_ = 8.838, *P* < 0.001]. **P* < 0.05 vs. the 12.5% risk probability group each day. ^#^*P* < 0.05 vs. the 25% risk probability group on each day, ^$^*P* < 0.05 vs. the 12.5% reward probability group on each day. ^†^*P* < 0.05 vs. the 25% reward probability group on each day. **(B)** Effect of changing the risk/reward probability on the number of entries into empty arms. There was no significant difference between the groups.

Supplementary Figure 3 **(A)** Effect of intra-AIC injection of ICI in control mice in decision-making task. **(B)** Effect of ICI on the number of entries into empty arms. There was no significant difference between the groups.
