# Supplementary figures and images for: Stress-altering anterior insular cortex activity affects risk decision-making behavior in mice of different sexes
Source: Front Cell Neurosci. 2023 Jan 24;17:1094808. doi: 10.3389/fncel.2023.1094808 (PMC9902351; doi:10.3389/fncel.2023.1094808)

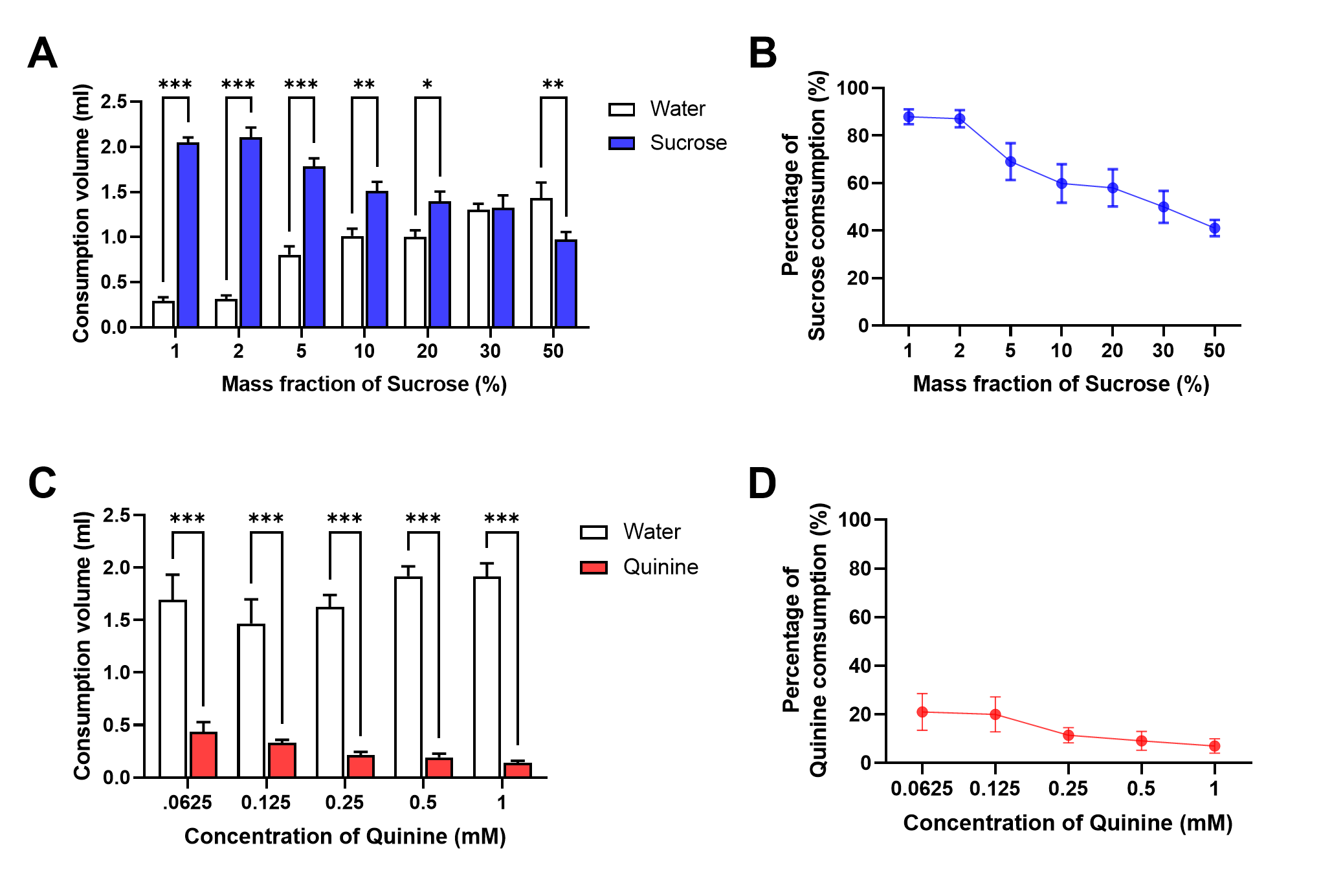

Supplement: Supplementary Figure 1 — (A) Effect of different mass fractions of sucrose solution on sucrose preference. During the habituation stage of the risk decision-making test, water and sucrose solutions were placed separately in the baited arm. The consumption volume was calculated in 10-min free exploration. (B) Percentage of sucrose consumption to the total solution consumed (%). (C) Effect of different mass fractions of quinine solution on quinine aversion. (D) Percentage of quinine consumption to the total solution consumed (%). *P < 0.05, **P < 0.01, ***P < 0.001 (two-way ANOVA with Bonferroni correction). [file Image_1.tif]

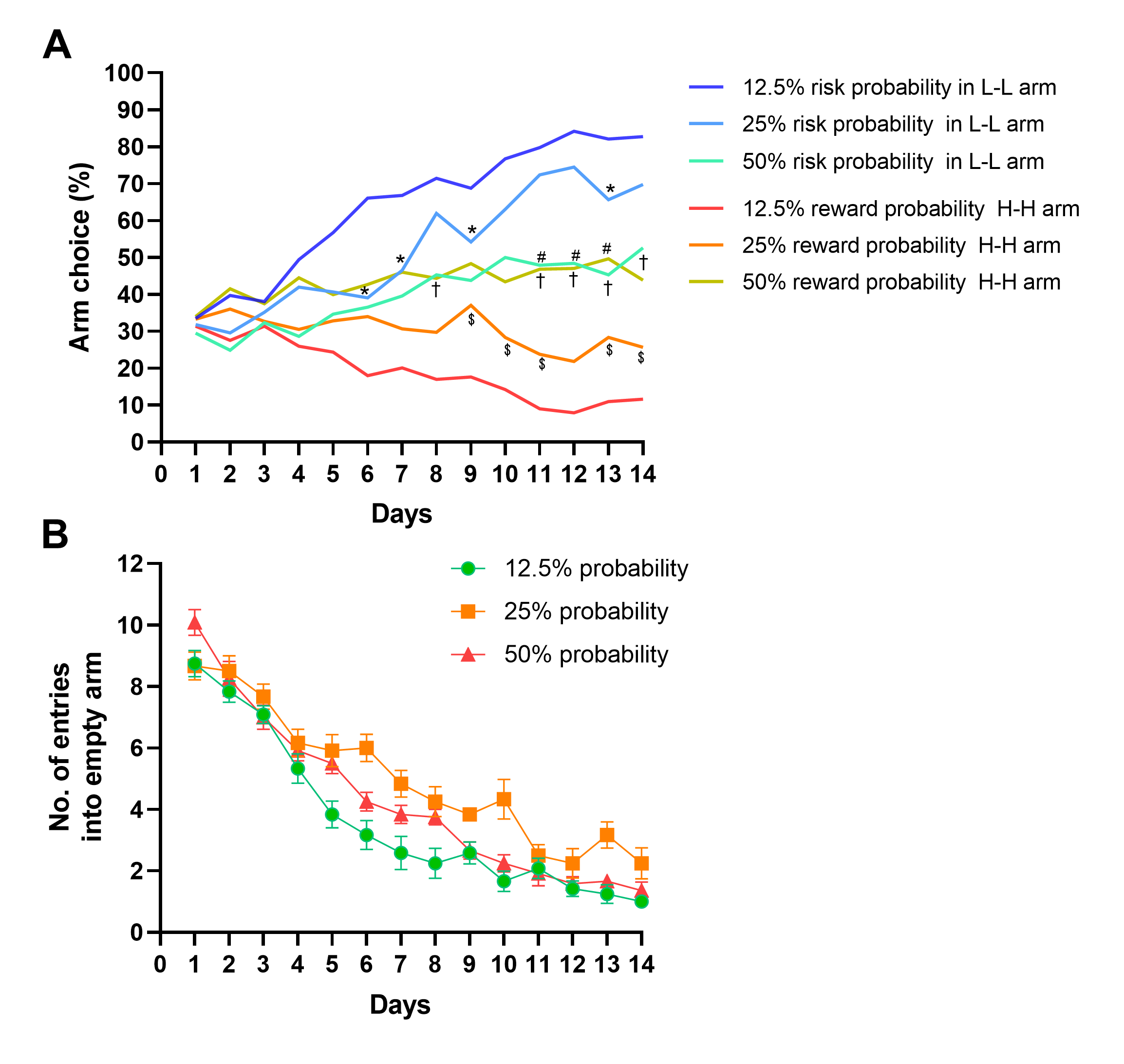

Supplement: Supplementary Figure 2 — (A) Effect of changing the probability of risk/reward on arm choice ratio in the mice gambling test. The risk and reward probability were increased from 12.5% to 50% simultaneously. Values are means ± SEM (n = 12). Repeated ANOVA revealed significant effects on risk/reward probability F(5,66) = 137.7, P < 0.001, time F(66,858) = 2.029, P < 0.001, and their interaction F(65,858) = 8.838, P < 0.001]. *P < 0.05 vs. the 12.5% risk probability group each day. #P < 0.05 vs. the 25% risk probability group on each day, $P < 0.05 vs. the 12.5% reward probability group on each day. †P < 0.05 vs. the 25% reward probability group on each day. (B) Effect of changing the risk/reward probability on the number of entries into empty arms. There was no significant difference between the groups. [file Image_2.tif]

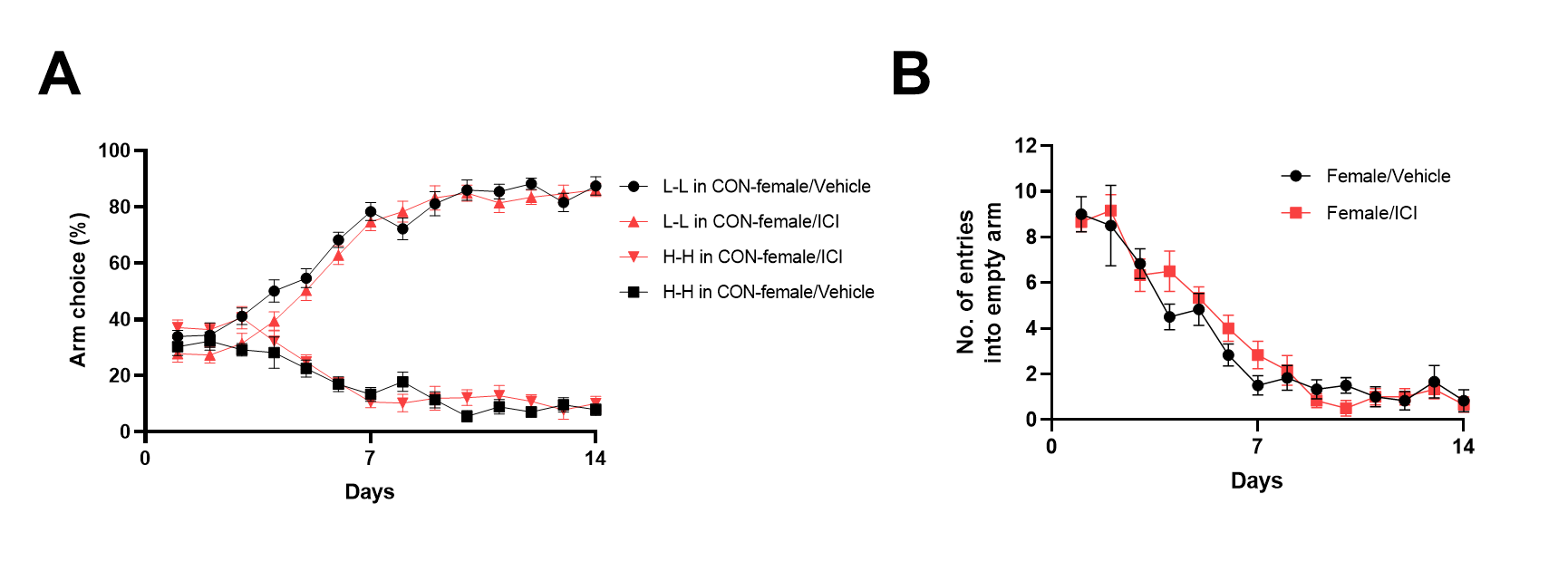

Supplement: Supplementary Figure 3 — (A) Effect of intra-AIC injection of ICI in control mice in decision-making task. (B) Effect of ICI on the number of entries into empty arms. There was no significant difference between the groups. [file Image_3.tif]
